# Editorial of the Special Issue: cGMP-Signaling in Cells and Tissues: Molecular, Functional and Pharmacological Aspects

**DOI:** 10.3390/ijms23126482

**Published:** 2022-06-10

**Authors:** Jens Schlossmann

**Affiliations:** Department of Pharmacology and Toxicology, Institute of Pharmacy, University of Regensburg, 93040 Regensburg, Germany; jens.schlossmann@chemie.uni-regensburg.de; Tel.: +49-941-943-4770; Fax: +49-941-943-4772

## 1. Communication

Several important and novel aspects regarding signaling by cGMP were reported in the various publications of this Special Issue. The topics comprise the regulation of cGMP synthesis by soluble guanylyl cyclase (sGC, NO-GC) and its structural features, signaling by cGMP-dependent protein kinases (PKG) and its substrates as well as the cGMP degradation and modulation by phosphodiesterases (PDE). The features of the diverse manuscripts and reviews of this Special Issue include possible (patho)physiological functions and pharmacological application of signaling by cGMP in a variety of syndromes comprising skeletal muscle, renal, cardiovascular, gastrointestinal, hematological and cancer diseases.

The modulation of sGC is currently emerging as a pharmacological tool in various diseases. The NO-independent and heme-dependent sGC stimulator riociguat was first introduced into therapy of pulmonary arterial hypertension and chronic thromoboembolic pulmonary hypertension [1]. Since then, there were several approaches to enlarge the pharmacological application of this class of drugs, which lead to the sGC stimulator vericiguat, a recent medication for chronic heart failure with reduced ejection fraction [2]. In the Special Issue, the manuscript by Krishnan et al. reports a potential further treatment option for sGC modulation in the context of Duchenne Muscular Dystrophy (DMD). The sGC stimulator BAY-747 improved several functional and pathological parameters of skeletal muscle in the DMD murine model, which suggests a possible application of sGC modulators in this disease [3].

A further class of sGC modulators are the nitric oxide- and heme-independent sGC activators. This class was not introduced into pharmacological applications up to now possibly because the sGC activator cinaciguat showed strong blood pressure reductions during clinical tests. However, there might be possible application of sGC activators in chronic diseases, e.g., diabetic nephropathy [4]. A novel aspect of pharmacological treatment would be the selective targeted delivery of the drug into specific cells. This principle was used by Fleischmann et al. The sGC activator cinaciguat was selectively targeted to renal mesangial cells by designed viral mimetic nanoparticles, which could offer novel possible treatment options for chronic kidney diseases and renal fibrosis [5].

New structural features of sGC are essential for understanding the precise molecular mechanism and modulation of this enzyme. In the review by Wittenborn et al. the current view of full length sGC revealed by novel structures obtained by cryo-electron microscopy is summarized. The authors discussed the inactivated and activated states of the enzyme to describe the detailed biochemical mechanism and its function, which could be helpful to elucidate new sGC modulators for pharmacological treatments [6].

Signaling by cGMP dependent protein kinases (PKG) and their substrates are important aspects for understanding the specific pathways and functions of these kinases in different physiological systems. New aspects regarding the role of cysteine-rich LIM-only protein 4 (CRP4) in cGMP signaling were investigated by Längst et al., which revealed that CRP4 acts as a feedback loop for relaxation of the vasculature [7].

The involvement of cGMP signaling deficiency in gastrointestinal and hematological disorders was reported by Majer et al. Loss of the IP_3_R-associated cGMP kinase substrate 1 (IRAG1) in mice exerts gastrointestinal disorders and bleeding, anemia, iron deficiency and splenomegaly. This is accompanied by downregulation of the specific β-isoform of PKGI (PKGIβ) protein. This leads to the hypothesis, that a loss of PKGIβ/IRAG1 signaling causes these physiological defects [8].

Phosphodiesterases (PDE) modulate the level of cyclic nucleotides in cells and are very effective drug targets. There are various PDE known, which are involved in diverse (patho)physiological functions. PDE2 is stimulated by cGMP and induces cAMP hydrolysis. In the manuscript by Wagner et al. PDE2 was overexpressed in mice and thereby protection against ventricular arrhythmia was observed [9]. Hence, the activation of myocardial PDE2 might lead to new therapy options in anti-arrhythmic therapy of heart failure.

cGMP-hydrolyzing phosphodiesterase (cGMP-PDE) are appealing targets for a variety of diseases and might be also valuable in the treatment of cancer. Di Iorio et al. reviewed the possible role of cGMP-PDE in breast cancer and discussed the potential application of cGMP-PDE inhibitors. For this, the notable and controversial studies concerning an off-label use of these inhibitors in cancer therapy are summarized [10].

## 2. Conclusions

Signaling by cGMP is an appealing topic in biochemistry, (patho)physiology and pharmacology. It is expected that the structural, biochemical and dynamic aspects of the signaling by cGMP network are further elucidated. Furthermore, there are several new treatment options of cGMP modulation arising in the future in various diseases. These various aspects of signaling by cGMP suppose that this attracting field will further extend in future.
